# Recovery and Stability of Omadacycline 150-mg Crushed Tablets Dispersed in Food or Water and Administered via Nasogastric Tube

**DOI:** 10.1093/ofid/ofag011

**Published:** 2026-02-17

**Authors:** Chenlin Hu, Ricky Huynh-Phan, Kevin M Rodriguez Robles, Taryn A Eubank, Kevin W Garey

**Affiliations:** Department of Practice and Translational Research, University of Houston College of Pharmacy, Houston, Texas 77204, USA; Department of Practice and Translational Research, University of Houston College of Pharmacy, Houston, Texas 77204, USA; Department of Practice and Translational Research, University of Houston College of Pharmacy, Houston, Texas 77204, USA; Department of Practice and Translational Research, University of Houston College of Pharmacy, Houston, Texas 77204, USA; Department of Practice and Translational Research, University of Houston College of Pharmacy, Houston, Texas 77204, USA

**Keywords:** crushing, epimer, nasogastric tube, omadacycline, recovery

## Abstract

**Background:**

Omadacycline, an aminomethylcycline tetracycline available orally and intravenously, is approved for the treatment of patients with acute bacterial skin and skin structure infections and community-acquired bacterial pneumonia, some of whom have difficulty swallowing tablets. The objective of this study was to assess the stability and recovery of crushed omadacycline 150-mg tablets.

**Methods:**

The recovery and stability of crushed omadacycline 150-mg tablets was assessed using 3 crushing methods (pill crusher, mortar/pestle, and sandwich bag method) and 3 dispersal products (water, vanilla syrup, or applesauce) over 24 hours at room temperature and administered via nasogastric tube. Omadacycline and its epimer (OMC-4-epimer) concentrations were assessed via liquid chromatography tandem mass spectrometry.

**Results:**

There was no significant difference in recovery of omadacycline or OMC-4-epimer proportion based on crushing technique. There were significantly higher proportions of OMC-4-epimer in applesauce experiments than in water or vanilla syrup experiments, regardless of crushing methods. All experiments had significantly less recovery of omadacycline and increased proportions of OMC-4-epimer at 24 hours compared with earlier time points. There was no significant difference in recovery of omadacycline or OMC-4-epimer proportion using either type of nasogastric tube with omadacycline recovery above 91%.

**Conclusions:**

The optimal method for recovery and stability of crushed omadacycline was to rinse the crushing device thoroughly into the collection container and disperse it into water, with consumption immediately or after no more than 4 hours. Vanilla syrup may be used as an alternative dispersal vehicle to be consumed immediately if used.

## Graphical Abstract

**Graphical Abstract:**
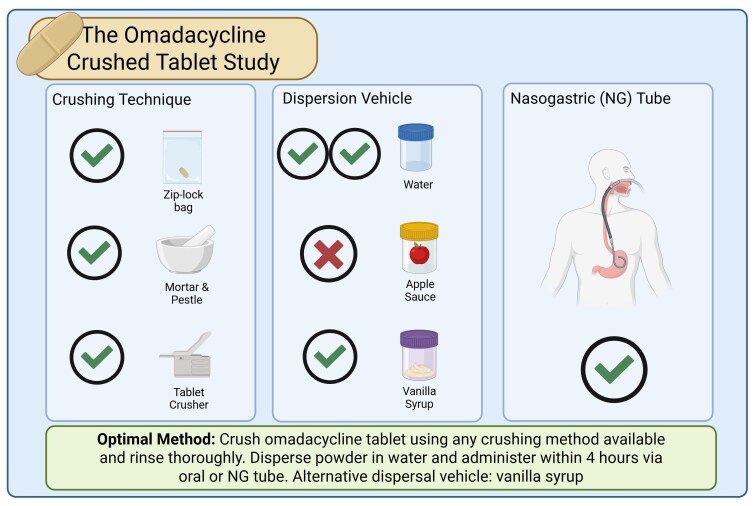

Omadacycline is an aminomethylcycline tetracycline-class antibiotic approved by the Food and Drug Administration to treat community-acquired bacterial pneumonia and acute bacterial skin and skin structure infections in adults [1]. It is formulated as a monotosylate salt and has both oral tablet and intravenous formulations [2]. Oral omadacycline has a relatively low bioavailability (approximately 35%); a 300-mg oral dose (2 omadacycline 150-mg tablets) is bioequivalent to a 100-mg intravenous dose [3]. Oral administration of the tablet is recommended for patients who can tolerate oral consumption [4]. However, approximately 16% of adults have dysphagia or trouble swallowing, making oral medication consumption challenging in the outpatient setting [5]. In addition, many hospitalized patients can receive nothing by mouth before or after surgery or when admitted to the intensive care unit, necessitating a nasogastric (NG) tube. To address this administration problem, a common practice is to first crush the tablet and then mix the drug tablet powder with liquid or soft food (eg, water or applesauce) before administration. For patients requiring an NG tube, the crushed tablet is administered in water [6]. While this is a useful strategy, the impact of crushing, dispersal vehicle choice, or NG tube administration on omadacycline stability and recovery is unknown.

In addition to low bioavailability and unknown stability when crushed, omadacycline, like other tetracyclines, undergoes spontaneous epimerization at the C-4 position and is converted to omadacycline-4-epimer (OMC-4-epimer) which has no or diminished susceptibility compared with omadacycline [7]. Epimerization is affected by environmental factors, such as pH [8]. Thus, the purpose of this study was to investigate the effects of 3 different crushing methods (pill crusher, mortar/pestle, and sandwich bag method), of 3 different dispersing vehicles (water, vanilla syrup, or applesauce) over 24 hours, and of administration via NG tube on the stability and recovery of omadacycline and degree of epimerization to OMC-4-epimer.

## METHODS

### Crushing Methods

Three separate methods of crushing 150-mg omadacycline tablets were tested. With the first method (pill crusher), the tablet (Paratek Pharmaceuticals) was placed in a pouch and crushed using the Silent Knight tablet crushing device (NONSK0100; Medline). With the second method (mortar and pestle), the tablet was placed into a mortar and crushed using the pestle. With the third method (sandwich bag), the tablet was placed in a plastic zipper-lock sandwich bag, and a weight was added on top of the bag to crush the tablet.

### Dispersal Vehicle Methods

A 150-mg omadacycline tablet was crushed using one of the methods described above, and the tablet powder was transferred into a 50-mL centrifuge tube and mixed well with 30 mL of the vehicle (water, Mott's applesauce, or Torani vanilla syrup). The powder-containing device was rinsed when applicable. To disperse tablet powder in the applesauce when using the pill crusher, the device was rinsed with an additional 10 mL of water. The tablet powder-vehicle mixture was incubated at room temperature in the dark for 0, 2, 4, or 24 hours.

### Omadacycline Recovery With Different Crushing or Dispersal Vehicle Methods

For all of the above experiments, the mixture in the 50 -mL centrifuge tube was inverted ≥6 times and mixed on the Vortex-Genie 2 Shaker (Scientific Industries) for 30 seconds. After centrifugation at 10 000*g* for 3–10 minutes (Centrifuge 5804 R; Eppendorf), a portion of supernatant (40 µL) was mixed well with water (1560 µL), yielding 40-fold dilution solution. Then a portion of the solution above (10 µL) was mixed well with extraction solvent (methanol-water–ethylenediaminetetraacetic acid disodium salt dihydrate [EDTA-Na_2_], 50% methanol/0.5 mol/L EDTA-Na_2_ [pH 8.0]; vol/vol 9/1; 90 µL) containing the internal standard, omadacycline-D9 (400 ng/mL), followed by further 100-fold dilution with 50% methanol. The final dilution solution was transferred into a vial and stored at −80°C prior to liquid chromatography tandem mass spectrometry (LC-MS/MS) analysis.

### NG Tube Method and Recovery Assessment

A 150-mg omadacycline tablet was crushed using the Silent Knight tablet crushing device, dispersed in water (30 mL), transferred into a 50-mL centrifuge tube, inverted, and vortexed as described above. Subsequently, the omadacycline suspension was transferred to a syringe that was connected to 1 of 2 types of NG tube, the Salem sump dual-lumen stomach tube (18F/Ch [6.0 mm] × 122 cm; reference 8888264986) or the Kangaroo NG feeding tube (10F × 109 cm; reference 8884-721088). The omadacycline-water suspension was passed through the tube and drained into a 100-mL volumetric flask, modified from previously published methods [9, 10]. The original 50-mL centrifuge tube containing omadacycline powder was rinsed twice with water (with 30 mL each time), which was poured into the syringe and drained into the 100-mL volumetric flask via the NG tube. After water was added to the volumetric flask to reach the 100-mL graduation mark, the flask was inverted ≥20 times. A portion of the water-omadacycline suspension (900 µL) was taken from flask and centrifuged at 10 000*g* for 3 minutes (Centrifuge 5804 R; Eppendorf), and a portion of supernatant (40 µL) was mixed with water (1560 µL), yielding a 40-fold dilution solution. Then a portion of the 40-fold dilution solution above (10 µL) was mixed well with internal standard (IS), omadacycline-D9-containing extraction solvent (90 µL), followed by further 100-fold dilution with 50% methanol. The final dilution solution was transferred into a vial and stored at −80°C prior to LC-MS/MS analysis.

### Patient Consent Statement

This study did not include factors necessitating patient consent.

### LC-MS/MS Analysis

Five microliters of an omadacycline tosylate calibration solution or a diluted sample from the omadacycline tablet experiments described above was analyzed in the SCIEX ExionLC liquid chromatography system coupled with a QTRAP 5500 mass spectrometer (SCIEX), according to our previously validated method (working range, 0.1–200 ng/mL) [7]. Because of spontaneous epimerization, omadacycline and its epimerization product, OMC-4-epimer, coexist in a sample. In this study, *total omadacycline* refers to the sum of omadacycline and OMC-4-epimer in a sample. The concentration of total omadacycline in a sample was measured from the response of a tested sample against the calibration curve, and the relative abundance (or proportion) of OMC-4-epimer in a tested sample was estimated by dividing the peak area of OMC-4-epimer by the sum of peak areas of omadacycline and OMC-4-epimer in the LC-MS/MS chromatogram.

### Statistical Analysis

All statistical analysis was performed using SAS software, version 9.2 (SAS Institute) or NCSS 2022 software (NCSS). The difference in recovery of total omadacycline (or the proportion of OMC-4-epimer) was analyzed using 2-way analysis of variance with Duncan multiple range test or Kruskal-Wallis or Student *t* tests when appropriate. Differences were considered significant at *P* < .05.

## RESULTS

### Impact of Crushing Method on Omadacycline Recovery and Stability

All experiments were done at least in duplicate (Tables 1 and 2). Immediate recovery of omadacycline or its OMC-4-epimer proportion from water or applesauce when the tablet as crushed using a pill crusher, mortar and pestle, or the sandwich bag method averaged 86% (6%) (mean [SD]) for omadacycline and 11% (1%) for its epimer (total recovery, 97% [6%]). Omadacycline recovery was similar using applesauce (mean [SD], 85% [4%]) or water (87% [7%]) and using different crushing techniques (pill crusher, 89% [6%]; mortar and pestle, 85% [5%]; sandwich bag, 86% [6%]). The OMC-4-epimer proportion was numerically higher in applesauce (mean [SD], 12% [0.7%]) than in water (10% [0.75%]) but similar for the 3 crushing techniques (pill crusher, 11% [0.6%]; mortar and pestle, 11% [1.2%]; sandwich bag, 11% [1.7%]). The results for omadacycline, OMC-4-epimer, and total omadacycline based on dispersal product and crushing method are shown in Table 1. There was no significant difference in recovery of omadacycline or OMC-4-epimer proportion based on crushing technique. There was a significantly higher proportion of OMC-4-epimer in applesauce experiments than to water experiments, regardless of the crushing method (*P* < .001). Omadacycline recovery was lower in applesauce experiments, but these differences were not statistically significant (*P* = .052).

**Table 1.** Recovery and Stability of Omadacycline (OMC) and Total OMC (OMC + OMC-4-Epimer) and Proportion of OMC-4-Epimer in Water or Applesauce With 3 Crushing Methods

Dispersal Product	Crushing Method	No. of Experiments	Mean (SD), %		
			OMC	OMC-4-Epimer	OMC + OMC-4-Epimer
Water	Pill crusher	3	91 (8)	11 (0.5)	102 (9)
	Mortar and pestle	6	82 (4)	10 (0.8)	91 (4)
	Sandwich bag	6	89 (6)	10 (0.7)	99 (6)
Applesauce	Pill crusher	3	86 (1)	12 (0.2)	97 (2)
	Mortar and pestle	6	87 (4)	12 (0.4)	100 (5)
	Sandwich bag	4	82 (4)	13 (0.8)	94 (6)

Abbreviation: OMC, omadacycline.

**Table 2.** Recovery and Stability of Omadacycline (OMC) and Total OMC (OMC + OMC-4-Epimer) and Proportion of OMC-4-Epimer in Water, Applesauce, and Syrup Over 24 Hours

Dispersal Product	No. of Experiments	Time, h	Mean (SD), %		
			OMC	OMC-4-Epimer	OMC + OMC-4-Epimer
Water	3	0	91 (8)	11 (0.5)	102 (9)
	3	2	93 (2)	12 (0.6)	106 (3)
	3	4	98 (4)	12 (0.06)	112 (4)
	3	24	84 (3)	15 (0.4)	98 (4)
Applesauce	3	0	86 (1)	12 (0.2)	97 (2)
	3	2	88 (4)	14 (0.7)	102 (4)
	3	4	86 (3)	17 (0.3)	105 (4)
	3	24	73 (0.1)	25 (0.1)	97 (0)
Vanilla syrup	2	0	98 (4)	11 (0.7)	110 (4)
	2	2	102 (1)	13 (1.2)	118 (2)
	3	4	90 (3)	14 (0.6)	105 (4)
	3	24	81 (5)	21 (0.5)	103 (6)

Abbreviation: OMC, omadacycline.

### Impact of Dispersal Vehicle on Omadacycline Recovery and Stability

Recovery of omadacycline ranged from 91% (7%) (mean [SD]) to 79% (6%) over 24 hours, while the OMC-4-epimer proportion ranged from 11% (0.6%) to 20% (4.5%) (total recovery, 99% [5%] to 107% [7%]). Omadacycline recovery was highest in water (mean [SD], 92% [7%]) or vanilla syrup (92% [9%]) than in applesauce (83% [7%]). The OMC-4-epimer proportion was lowest in water (mean [SD], 12% [2%]) followed by vanilla syrup (15% [4%]) and applesauce (17% [5%]). Results for omadacycline, OMC-4-epimer, and total omadacycline based on dispersal product and time are shown in Table 2. Significant results of the analysis of variance Duncan comparisons (*P* < .05) showed significantly less recovery of omadacycline with applesauce than with water or syrup, and significantly increased proportions of OMC-4-epimer with applesauce than with syrup, which also had significantly increased proportions of OMC-4-epimer compared with water. All experiments had significantly less recovery of omadacycline and increased proportions of OMC-4-epimer at 24 hours compared with earlier time points. The interaction term between applesauce and the 24-hour time point was also significant, predicting lower omadacycline recovery and a higher OMC-4-epimer proportion.

### Impact of NG Tube Administration on Omadacycline Recovery and Stability

Recovery of omadacycline after passage through 1 of 2 NG tubes ranged from 91% to 96%, and the proportion of OMC-4-epimer ranged from 9% to 14% (total recovery, 102%–108%). Results from 3 replicate experiments using the Salem sump dual-lumen stomach or the Kangaroo NG feeding tube are shown in Table 3. There was no significant difference in recovery of omadacycline or OMC-4-epimer between the 2 NG tubes, with omadacycline recovery >91% for all experiments.

**Table 3.** Recovery and Stability of Omadacycline (OMC) and Total OMC (OMC + OMC-4-Epimer) and Proportion of OMC-4-Epimer in Water Administered Through Nasogastric Tubes, With Results of 3 Individual Experiments for Each Tube

NG Tube	Experiment No.	OMC, %	OMC-4-Epimer, %	OMC + OMC-4-Epimer, %
Kangaroo NG feeding tube	1	93	9	102
	2	96	11	108
	3	91	14	106
Salem sump dual-lumen stomach tube	1	94	10	105
	2	93	11	105
	3	92	11	103

Abbreviations: NG, nasogastric; OMC, omadacycline.

## DISCUSSION

For patients unable to swallow oral tablets, it is common to crush solid oral medications and disperse them into water or certain foods to facilitate administration, including NG tube administration. Before the current study, no information was available regarding the stability and recovery of omadacycline when crushed and dispersed into a dispersal vehicle or passed through an NG tube. We tested multiple methods of crushing and found that the method did not affect stability or recovery. For each crushing method, we noticed a small amount of residual powder after crushing. For this reason, we rinsed each crushing container with a small amount of water as part of our scientific method. This procedure should be considered if used clinically to ensure that all crushed omadacycline powder is transferred into the dispersal vehicle. Crushed omadacycline has a noticeable yellowish color, making the assurance of proper rinsing straightforward.

Tetracyclines, including omadacycline, have a known drug interaction with calcium and other divalent cations [11]. Chelation of tetracyclines with divalent cations forms an insoluble product that limits absorption in the gastrointestinal tract. For this reason, crushed tetracycline tablets should never be dispersed in milk or other foods high in calcium or other divalent cations. We chose applesauce and vanilla syrup as our food dispersal products in this study due to the low amount of divalent cations contained in these products. Despite this, we saw a significantly lower proportion of omadacycline recovered from applesauce experiments in our time studies and a numerically lower proportion recovered in our crushing studies. Although the quantities present are not large enough to improve human health, applesauce can contain up to 10 mg of calcium and other divalent cations per 1-cup serving [12]. It is possible that the small amounts of calcium present in applesauce reduced the proportion of recovery of omadacycline in our experiments. Tetracyclines also readily form epimers, especially under acidic conditions [13].

In our prior human, healthy-volunteer study, our group demonstrated that the OMC-4-epimer averaged approximately 9% of total omadacycline in vitro in fecal matrix and increased to 37% in human fecal samples [7]. Both applesauce and vanilla syrup are weakly acidic products. In our time experiments we saw significantly increased proportions of the OMC-4-epimer at the 24-hour time point. We recommend that omadacycline be crushed using a convenient method, with the container rinsed after crushing. Water is the preferred dispersal product, with vanilla syrup recommended for people who need a slightly sweeter taste. The crushed and dispersed omadacycline should be ingested as soon as possible and no later than 4 hours after dispersing the crushed tablet into the dispersal vehicle. Our results using the NG tubes are concordant with these recommendations, as the crushed omadacycline tablet was dispersed in water and immediately put through the NG tube with good omadacycline recovery.

This study has certain limitations. We performed in vitro experiments only and did not assess the effects of crushing and dispersal products on human pharmacokinetics or treatment of infections. Two of our vanilla experiments failed (at the 0- and 2-hour time points), so these experiments were performed only in duplicate. In addition, the safety and efficacy of crushed omadacycline tablets in clinical use is unknown and warrants further investigation. We demonstrated higher proportions of the OMC-4-epimer in applesauce and vanilla syrup at later time points, which we hypothesize is due to the low acidity of these products. Because epimerization of omadacycline spontaneously occurs [7], omadacycline and OMC-4-epimer coexist in aqueous samples or human biologic samples. However, OMC-4-epimer’s susceptibility to microorganisms and potential anti-inflammatory properties should be tested in the future. Immediate dispersal and consumption will minimize epimerization.

In conclusion, the total in vitro recovery of omadacycline and its OMC-4-epimer was excellent regardless of the crushing method, the dispersal product, or the NG tube. Rinsing the crushing device thoroughly into the collection container, dispersing the powder into water, and consuming the crushed tablet immediately or after no more than 4 hours provided optimal results in our experiments. Vanilla syrup is an alternative dispersal vehicle to be consumed immediately if used clinically.
